# Integrative In Silico Analysis of miRNA–mRNA Regulatory Networks in the Prefrontal Cortex of Individuals with Psychiatric Disorders Who Died by Suicide

**DOI:** 10.3390/ijms27073126

**Published:** 2026-03-30

**Authors:** José Luis Cortéz-Sánchez, Hernán Mauricio Rivera-Escobar, Esther Natalia Muñoz Roa, Carlos Andrés Zabala-Bello, Gilberto Pérez-Sánchez, José Miguel Chin Chan, Monserrat Bautista-Ortiz, Karla María López-Martínez, Federico Osorio-Antonio, José Luis Gálvez-Romero, Alan Carrasco Carballo, Virginia Sedeño-Monge, Francisco Castelán, Elizabeth Bautista-Rodríguez

**Affiliations:** 1 Environmental Epigenetics and Mental Health Laboratory, Faculty of Chemical Biological Sciences, Autonomous University of Campeche, Campeche 24085, Mexico; jose.cortez@udlap.mx (J.L.C.-S.); josmchin@uacam.mx (J.M.C.C.); 2 Health Sciences, University of the Americas, Puebla (UDLAP), Puebla 72810, Mexico; 3 Research Group on Biosciences, Pedagogy, and Socio-Environmental Sciences BIOPESA, University of Tolima, Ibagué 730006, Colombia; hmauriciore@ut.edu.co; 4 PhD Program in Biological Sciences, Pontificia Universidad Javeriana, Bogotá 110211, Colombia; en.munozr@javeriana.edu.co; 5 Animal Science and Veterinary Medicine Faculty, Universidad Nacional de Colombia, Bogotá 15372, Colombia; caazabalabe@unal.edu.co; 6 Psychoimmunology Laboratory, Neuroscience Department, Ramón de la Fuente Muñiz National Institute of Psychiatry, Mexico City 14370, Mexico; gilberto.perez.sanchez.1981@gmail.com; 7 Centro Tlaxcala de Biología de la Conducta, Universidad Autónoma de Tlaxcala, Tlaxcala 90062, Mexico; monse.ortbau@gmail.com (M.B.-O.); fcocastelan@iibiomedicas.unam.mx (F.C.); 8 Independent Researcher, Puebla 72410, Mexico; 9 Hospital Infantil de Tlaxcala (HIT), Tlaxcala 90606, Mexico; federico.osorio@upaep.edu.mx; 10 Hospital Regional del ISSSTE, Puebla 72570, Mexico; jose.galvez@issste.gob.mx; 11 SECIHTI, LESQO Adscrito a la Benemérita Universidad Autónoma de Puebla, Puebla 72590, Mexico; alan.carrascoc@correo.buap.mx; 12 Decanato de Ciencias Médicas, Universidad Popular Autónoma del Estado de Puebla (UPAEP)-SECIHTI-Puebla, Puebla 72410, Mexico; 13 Instituto de Investigaciones Biomédicas, Universidad Nacional Autónoma de México, Mexico City 04510, Mexico; 14 Multidisciplinary Laboratory in Biomedicine, Biotechnology and Integrative Bioinformatics Applied to Health (LAMB3IS), Faculty of Health Sciences, Autonomous University of Tlaxcala (UATx), Zacatelco 90750, Mexico; 15 Institut de Pharmacologie et de Biologie Structurale (IPBS-CNRS), 31077 Toulouse, France

**Keywords:** suicide, mRNA, miRNAs, regulatory network, biomarkers

## Abstract

To explore the regulatory aspects of mRNAs and miRNAs in suicide, we integrated transcriptomic data from GEO datasets. The analysis of mRNA expression in the prefrontal cortex of suicide victims with major depressive disorder revealed a differential profile with 27 downregulated mRNAs, including *HSPA1A*, *HSPA1B*, *DNAJB1*, *NR4A1*, and *GADD45B*, which are involved in proteostasis, transcriptional regulation, and apoptosis. Functional enrichment analysis using KEGG and Gene Ontology (GO) revealed significant associations with synaptic plasticity, neuronal survival, and signaling pathways, including MAPK, TGF-β, Wnt, p53, and neurotrophins. Subsequently, using the GSE34120 GEO dataset of miRNAs from the frontal cortex of suicide victims, 105 dysregulated miRNAs were identified. The networks revealed compact regulatory modules with hsa-miR-576-3p, hsa-miR-493, and hsa-miR-550, as well as highly connected central nodes such as hsa-miR-30b, hsa-miR-16a-5p, hsa-miR-181a-5p, and hsa-miR-184. The integration of both profiles allowed the elaboration of miRNA–mRNA regulatory networks in which *TP53*, *FOXO3*, *RELA*, and *FOS* interact with multiple dysregulated miRNAs. These findings support the notion that suicide involves complex post-transcriptional dysregulation, particularly related to astrocytic function and neurotrophic signaling, with potential diagnostic and therapeutic applications.

## 1. Introduction

Suicide is the fourth leading cause of death among young people aged 15 to 19 worldwide; 73% of global suicides occur in low- and middle-income countries [1]. The etiology of suicidal behavior is complex and multifactorial; among the most consistently associated factors are psychiatric illnesses. Mann and colleagues (1999) reported that approximately 95% of suicide victims had a mental illness such as depression, bipolar disorder, schizophrenia, anxiety, obsessive–compulsive disorders, post-traumatic stress disorder, substance abuse, dementia, brain trauma, and eating disorders [2]. From a neurobiological perspective, a diathesis–stress model has been proposed, in which environmental stressors interact with predisposing pathophysiological traits such as impulsivity and aggression, potentially triggering suicidal crises independently of specific psychiatric diagnoses [3]. Additionally, hyperactivity of the hypothalamic–pituitary–adrenal (HPA) axis has been associated with suicide, leading to elevated cortisol levels [4]. Suicide has also been related to low cerebrospinal fluid levels of the serotonin metabolite 5-hydroxyindoleacetic acid (CSF 5HIAA), polymorphisms in the 5-HT2A receptor gene and the serotonin transporter (5-HTTLPR), as well as reduced norepinephrine (NE) levels and decreased binding affinity to α2-adrenergic receptors in the prefrontal cortex [5,6]. Although these findings have contributed to understanding the neurobiological basis of suicidal behavior, key molecular mechanisms remain insufficiently characterized, particularly those involving gene expression regulation at both transcriptional and post-transcriptional levels. MicroRNAs (miRNAs) are small non-coding RNAs that regulate gene expression post-transcriptionally by binding to target mRNAs, thereby inhibiting translation or promoting mRNA degradation [7]. Growing evidence highlights their role in psychiatric disorders and in biological processes involved in behavioral regulation, suggesting that modulation of miRNA activity may represent a potential therapeutic strategy [7]. Additionally, miRNAs have been proposed as putative biomarkers of major depressive disorder and suicide-related behavior [8].

Several specific miRNAs have been implicated in mood disorders and stress-related conditions. For example, miR-124 and miR-132 have been associated with synaptic plasticity and stress-responsive signaling; miR-16 and miR-135a are linked to serotonergic regulation and antidepressant response; and members of the miR-34 family have been reported as dysregulated in depressive and stress-related phenotypes [7,8]. These findings support the concept that miRNA-mediated post-transcriptional regulation may contribute to molecular vulnerability in suicide [7,8]. In this context, the present study analyzed transcriptomic data from two independent postmortem cohorts. Initially, differential expression analysis of dataset GSE101521, which includes RNA-seq profiles from the dorsolateral prefrontal cortex (dlPFC; BA9) of individuals with major depressive disorder who died by suicide and non-psychiatric controls, revealed dysregulation of MAPK and neurotrophin signaling pathways [9]. These pathways are essential for neuronal survival and synaptic plasticity, suggesting a potential disruption of neurotrophic regulation in the dlPFC.

Given that brain-derived neurotrophic factor (BDNF) is a central regulator of synaptic plasticity and neuronal resilience, and considering that reduced BDNF levels have been consistently reported in both serum and frontal cortex samples of suicide victims [10,11], we further explored a biologically defined neurotrophic-related subgroup. For this purpose, we incorporated dataset GSE34120 [12], characterized by reduced expression of the TrkB-T1 receptor isoform. TrkB-T1 is a truncated isoform of the BDNF receptor predominantly expressed in astrocytes [13], and its reduced expression has been consistently observed in the brains of suicide victims [14]. Because TrkB-T1 modulates neurotrophic tone and synaptic homeostasis, alterations in this receptor may influence downstream MAPK signaling and related transcriptional programs. Rather than performing subject-level integration across independent cohorts, we applied a knowledge-based in silico regulatory overlap approach to explore potential convergence between independently derived mRNA and miRNA signatures in closely related prefrontal cortical regions. This strategy enabled the identification of regulatory hubs potentially linking altered TrkB–MAPK signaling with stress-responsive transcriptional pathways. We hypothesized that coordinated dysregulation of stress-responsive mRNA and miRNA-mediated post-transcriptional control in the prefrontal cortex of individuals who died by suicide may converge on BDNF/TrkB–MAPK-related neurotrophic signaling pathways, potentially contributing to altered synaptic plasticity and increased neurobiological vulnerability.

## 2. Results

The differential expression analysis of the miRNAs and mRNAs was conducted using the GEO database, including the datasets GSE34120 (frontal cortex) and GSE101521 (dorsolateral prefrontal cortex), respectively, confirmed by LIMMA [15]. From that analysis, the differentially expressed miRNAs and mRNAs were identified in brain tissue samples from suicide victims and control samples.

### 2.1. mRNAs Differentially Expressed in the Brain Tissue of Suicide Victims

**Figure 1:**
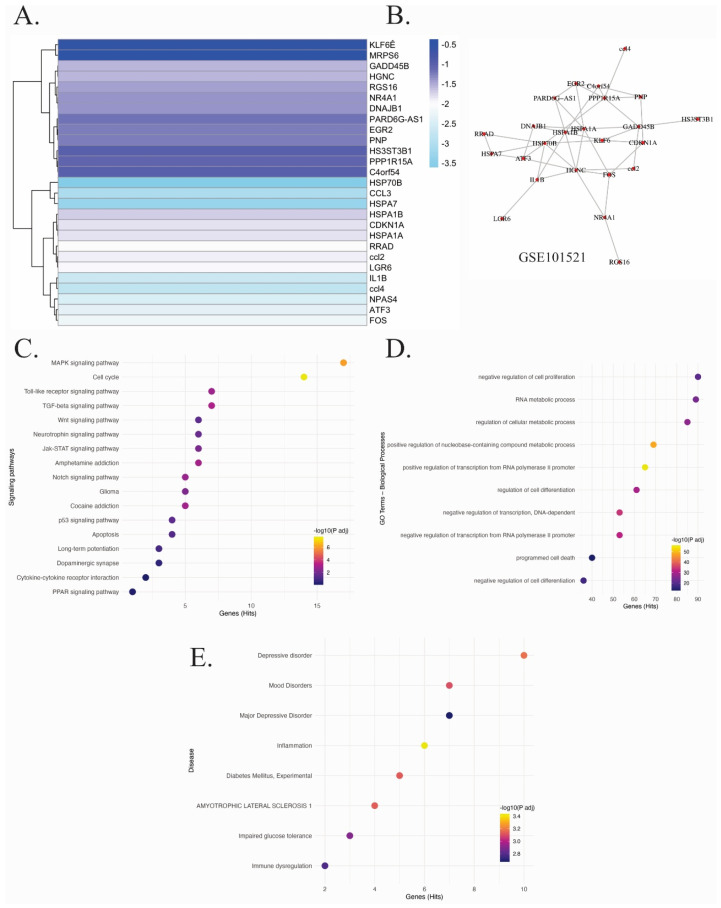
Differentially expressed mRNAs and associated molecular networks in the suicidal prefrontal cortex. Data from the GSE101521 dataset (dorsolateral prefrontal cortex, BA9) were gathered to perform a differential expression analysis of mRNAs. (**A**) Heatmap showing fold change values of differentially expressed mRNAs identified using DESeq2 compared with controls. (**B**) A co-expression (unweighted) network was constructed from the expression patterns of differentially expressed mRNAs. Nodes represent genes; edges, represent the statistically significant co-expression relationships identified using mutual information. Functional enrichment analysis based on KEGG pathways (**C**), Gene Ontology biological processes (**D**), and disease associations (**E**). The enrichment analyses were performed using an expanded network derived from the original differentially expressed genes. “Genes (Hits)” indicates the number of genes from this expanded network associated with each functional term. Statistical significance was set at *p* < 0.05, adjusted by using a hypergeometric test with multiple testing correction.

The study investigated mRNAs with altered expression in the dorsolateral prefrontal cortex (BA9) of suicide victims using the dataset GSE101521. A heatmap of differentially expressed genes (Figure 1A) revealed the downregulation of 27 mRNAs, including *KLF6e*, *MRPS6*, *GADD45B*, *HGNC*, *RGS16*, *NR4A1*, *DNAJB1*, *PARDEG-AS1*, *EGR2*, *HPN*, *H3SS3TB1*, *PPP1R15A*, *C4orf54*, *HSP70B*, *CCL3*, *HSPA7*, *HSPA1B*, *CDKN1A*, *HSPA1A*, *RRAD*, *CCL2*, *LGR6*, *IL1B*, *CCL4*, *NPAS4*, *ATF3*, and *FOS*. These genes are involved in cellular stress response, immune regulation, transcriptional control, and apoptosis [16,17,18,19,20]. In addition, *HSPA1A*, *HSPA1B*, and *DNAJB1* (members of the heat shock protein family) are crucial for maintaining proteostasis under stress conditions [21,22,23,24]. Their reduced expression suggests impaired protein folding and diminished cytoprotective capacity in the suicidal brain [21,22,23,24]. Other relevant downregulated transcripts include *NR4A1*, *GADD45B*, *EGR2*, and *PPP1R15A*, which are associated with transcriptional regulation, cell cycle control, and apoptosis [18,19,25]. Subsequently, we generated a gene interaction network via co-expression analysis, providing an integrated view of plausible functional interactions in the suicidal brain (Figure 1B). The enrichment analysis of biological processes (Figure 1D) showed prominent alterations in the negative regulation of transcription from RNA polymerase II promoter, programmed cell death, RNA metabolic processes, and cell differentiation and proliferation, while KEGG pathway enrichment (Figure 1C) highlighted several significantly affected signaling pathways, such as MAPK, TGF-beta, Wnt, p53, neurotrophin signaling, and JAK-STAT and cytokine-related pathways. These are fundamental for neuroplasticity, mood regulation, immune modulation, and survival, which align with the pathophysiological mechanisms of major depressive disorder and suicidal behavior. Disease enrichment analysis (Figure 1E) further supported the relevance of these findings, showing statistically significant associations with major depressive disorder, mood disorders, inflammation, diabetes mellitus, and amyotrophic lateral sclerosis (ALS).

### 2.2. miRNAs Differentially Expressed in the Brain Tissue of Suicide Victims

**Figure 2:**
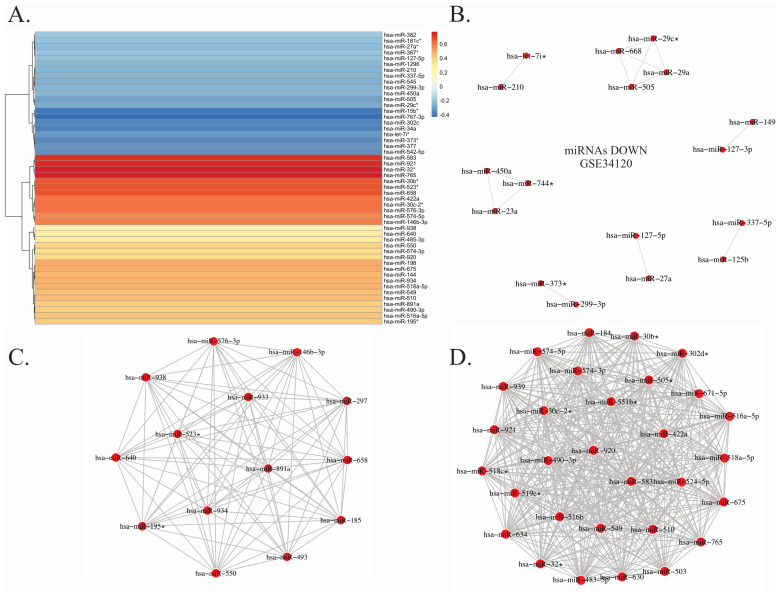
Differentially expressed miRNAs and associated molecular networks in the suicidal frontal cortex. Data from the GSE34120 dataset (frontal cortex) were gathered to perform a differential expression analysis of miRNAs. (**A**) Heatmap showing fold change values of the top 50 differentially expressed miRNAs identified using LIMMA compared with controls. (**B**–**D**) Co-expression networks were constructed from the expression patterns of downregulated and upregulated differentially expressed miRNAs. Nodes represent miRNAs, and edges correspond to statistically significant co-expression relationships identified using mutual information. Networks are visualized as unweighted graphs. The asterisk (*) denotes the non-coding strand derived from the miRNA precursor, which has historically been identified as the least abundant.

From the microarray dataset GSE34120, we identified 105 significantly dysregulated miRNAs in the frontal cortex of suicide victims with downregulated expression of TrkB-T1 (Figure 2A). Among them were ten significantly downregulated miRNAs (*p* ≤ 0.05) that regulate neurodevelopment, inflammation, and synaptic plasticity: hsa-miR-15b*, hsa-miR-767-3p, hsa-miR-505, hsa-miR-373*, hsa-miR-377, hsa-miR-29c*, hsa-miR-545, hsa-miR-302c, hsa-miR-139-5p, and hsa-miR-125b. Such a collective downregulation suggests a broad impairment in post-transcriptional gene regulation relevant to suicide neurobiology. The co-expression network of the downregulated miRNAs (Figure 2B) revealed several central nodes, such as the miR-29 family (miR-29a, miR-29c), miR-127-3p/5p, miR-149, and miR-125b. In contrast, the following 10 miRNAs were significantly upregulated (*p* < 0.05): hsa-miR-32*, hsa-miR-765, hsa-miR-583, hsa-miR-921, hsa-miR-30c-2*, hsa-miR-550, hsa-miR-574-5p, hsa-miR-576-3p, hsa-miR-198, and hsa-miR-503. From the two resulting co-expression networks of overexpressed miRNAs, the findings delineated one compact regulatory module (Figure 2C) comprising hsa-miR-576-3p, hsa-miR-493, and hsa-miR-550 as the nodes. Moreover, a denser and more complex network was also observed, in which miRNAs such as hsa-miR-30b, hsa-miR-16a-5p, hsa-miR-181a-5p, and hsa-miR-184 acted as highly connected central hubs (Figure 2D).

**Figure 3:**
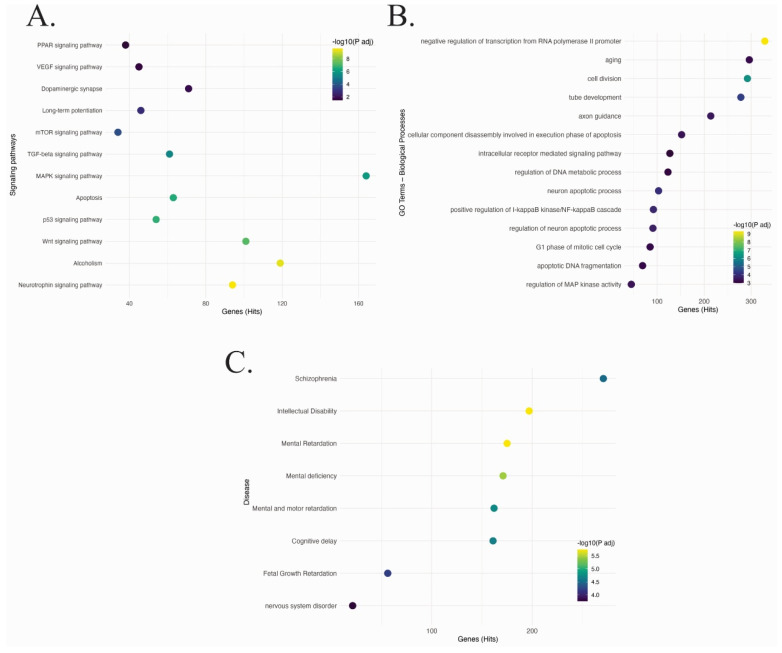
Functional enrichment analysis of differentially expressed miRNAs in the suicide frontal cortex (GSE34120 dataset, BA10). Functional enrichment analysis by KEGG signaling pathways (**A**), Gene Ontology biological processes (**B**), and disease associations (**C**), based on the differentially expressed miRNAs. All pathways and GO terms were considered statistically significant at a *p* < 0.05, adjusted by using a hypergeometric test with multiple testing correction.

A further exploration of functional implications was assessed via enrichment analysis conducted on predicted and validated gene targets using KEGG and GO databases (Figure 3). The resulting KEGG pathways included VEGF, PPAR, mTOR, MAPK, Wnt, and p53 signaling, as well as dopaminergic synapse and long-term potentiation, which are essential for emotional regulation and plasticity. Both mTOR and MAPK pathways are particularly implicated in suicide-related changes in neuroplasticity and the cellular stress response [9,10,11,12,13,14]. Gene Ontology analysis (Figure 3B) showed axon guidance, neuronal apoptosis, DNA fragmentation, and intracellular signaling cascades as enriched biological processes, suggesting compromised neurodevelopmental programs in suicide victims [7,8,26,27,28]. Moreover, disease enrichment analysis (Figure 3C) revealed strong associations with schizophrenia, intellectual disability, cognitive delay, fetal growth restriction, and other nervous system disorders.

### 2.3. Integrated miRNA–mRNA Regulatory Network in Suicide

**Figure 4:**
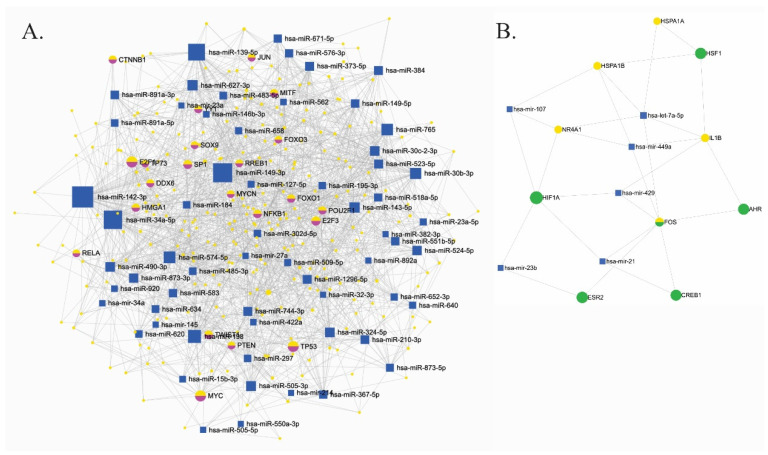
Integrated regulatory module of microRNAs (miRNAs), messenger RNAs (mRNAs), and transcription factors (TFs). (**A**) Complete regulatory module showing interactions among differentially expressed miRNAs (blue squares), their target genes (purple circles), and enriched transcription factors (yellow circles). The network was constructed using miRNet 2.0 based on the GSE34120 dataset and KEGG enrichment of the neurotrophin signaling pathway. (**B**) Simplified subnetwork highlighting transcription factors (green circles), differentially expressed genes (yellow circles), and regulatory miRNAs (blue squares) in the context of depression. Node size represents degree centrality (number of connections). Edges represent experimentally validated regulatory interactions and are unweighted.

We constructed a regulatory network of downregulated miRNAs and their validated mRNA targets using miRNet (Figure 4). A comprehensive network of miRNA–mRNA–TF interactions revealed multiple highly connected nodes, including *TP53*, *FOXO3*, *MYC*, *RELA*, *NFKB1*, *JUN*, *E2F3*, *HIF1A*, *PTEN*, and *HMGA1* (Figure 4A). Several miRNAs emerged as potential master regulators, including hsa-miR-145-5p, hsa-miR-27a-3p, hsa-miR-410, hsa-miR-485-3p, and hsa-miR-543, all of which are differentially expressed and predicted to regulate multiple nodes within the network. Figure 4B shows a functional subnetwork centered on *FOS*, one of the most downregulated transcripts identified in the GSE101521 dataset. In this subnetwork, FOS interacts with key molecules, including *CREB1*, *HIF1A*, *AHR*, and *ESR2*. This subnetwork also showed that *FOS* is regulated by hsa-miR-21 and hsa-miR-429, while *IL1B*, a pro-inflammatory cytokine, is targeted by let-7a-5p. *HSPA1A* and *HSPA1B*, members of the heat shock protein family, are regulated by hsa-miR-107.

## 3. Discussion

In this study, we integrated mRNA and miRNA expression profiles from the dorsolateral prefrontal cortex of individuals who died by suicide and non-psychiatric controls. Our results indicate a convergent dysregulation of immune and inflammatory pathways, cellular stress responses, and post-transcriptional networks involving miRNAs previously associated with neurodegeneration, ischemia, schizophrenia, and glioma. Overall, these findings support a multifactorial molecular substrate for suicidal behavior, with a prominent astrocytic and neuroimmune component.

We identified chemokines such as *CCL2*, *CCL3*, and *CCL4*, as well as the pro-inflammatory cytokine *IL-1β*, which are implicated in major depressive disorder and related conditions [29,30,31,32]. An increase in *CCL2* expression in the anterior cingulate cortex and hippocampus has been reported in depressed and suicidal patients [33,34]. Similarly, high levels (mRNA and protein) of *IL-1β* were detected in the prefrontal cortex of depressed individuals who died by suicide [16,35]. Several studies suggest that the increased levels of inflammatory cytokines observed in mood disorders occur in response to increased circulating mitochondrial DNA [36]. Moreover, a longitudinal study showed that plasma *IL-1β* is increased in late-life depression (LLD) patients compared with healthy controls, which was attributed to increased circulating mtDNA instability [37]. Therefore, the present data are consistent with a neuroinflammatory phenotype in the prefrontal cortex of suicide victims.

The analysis of mRNA expression showed alterations in members of the *HSP70* and *HSP40* heat shock protein families (e.g., *HSPA1A*, *HSPA1B*, *HSPA7*, *DNAJB1*) that are associated with schizophrenia and bipolar disorder [21,22,23,24]. Given their role in proteostasis and stress responses [21,22], their dysregulation may impair cellular resilience and promote an oxidative stress–inflammation interaction in the suicidal brain. However, it should be noted that acute cellular stress associated with agonal or perimortem processes could induce the expression of heat shock proteins, constraining an exclusive suicide-related context.

Furthermore, present findings showed alterations in the expression of transcription factors like *NPAS4*, *KLF6*, *NR4A1*, *EGR2*, *ATF3*, and *FOS*, considered key regulators in synaptic plasticity, excitatory–inhibitory balance, and stress and inflammatory responses [26,38,39]. NPAS4 is decreased in multiple brain regions of patients with major depression and suicide [25,40], while both NR4A1 and ATF3 are associated with chronic stress responses, including the pro-inflammatory monocyte profile, in severe psychiatric disorders [41,42]. *FOS* and *ΔFosB* have been described as central nodes in transcriptional networks related to depression, addiction, and suicide [43,44,45,46]. Nevertheless, several of these genes belong to the class of immediate early genes (IEGs), whose expression is rapidly induced by acute neuronal activation and stress [25,26,38]. Consequently, their altered expression in postmortem tissue may partly reflect terminal physiological responses or agonal factors rather than stable disease-related transcriptional programs. Genes such as *GADD45B*, *RGS16*, *CDKN1A*, *LGR6*, and *PNP*, each involved in neuroplasticity, *GPCR* signaling, cell cycle regulation, and purine metabolism, were also dysregulated. Many of these genes show relevant expression in astrocytes, supporting the hypothesis of an important glial contribution to the pathophysiology of suicidal behavior [17,18,19,20].

Present findings revealed the dysregulation of 105 miRNAs in the frontal cortex, with the following ten being significantly downregulated: hsa-miR-15b, -767-3p, -505, -373, -377, -29c, -545, -302c, -139-5p, and -125b. Six of them, miR-15b, miR-373, miR-29c, miR-545, miR-125b, and miR-505, have been linked to Alzheimer’s or Parkinson’s disease and proposed as biomarkers of neurodegeneration [47,48,49,50,51,52,53]. The miR-29c negatively regulates *BACE1*, implying its reduction favors the β-amyloid production [54,55]. The miR-125b participates in Hippo and PI3K–Akt pathways, axon guidance, and cytokine signaling, integrating synaptic and inflammatory signaling [56]. The miR-373 is decreased in neuronal exosomes from patients with moderate Alzheimer’s disease and exerts anti-inflammatory and anti-proliferative effects in other contexts by modulating P2X7R, IL-6, IL-8, CD44, and TGFBR2 [57,58]. Moreover, elderly patients with major depressive disorder show hsa-miR-184 expression levels lower than matched controls; however, this effect was not found, suggesting that age may play an important role [27]. Overall, the simultaneous downregulation of miRNAs herein suggests a molecular footprint similar to that observed in neurodegenerative processes, which agrees with a systematic review reporting miRNAs that are shared between MDD and Alzheimer’s disease [28].

The downregulation of a different subset of miRNAs could inform on ischemia and DNA repair, as well as other processes like neurodevelopment, synaptic plasticity, apoptosis, and inflammation. A decrease in miR-377 expression levels, as reported in oxygen–glucose deprivation models, can modulate endothelial survival and angiogenesis via *SIRT1*, *VEGFA*, and *BCL-XL* [59]. The miR-302c-3p, acting as a tumor suppressor in gliomas, has been proposed as a prognostic biomarker [60]. The miR-767-3p influences *MGMT* expression in glioblastoma, in association with the activation of TGF-β/PI3K–Akt pathway [61]. In contrast, the miR-139-5p, which is elevated in exosomes from patients with major depressive disorder and described as a negative regulator of hippocampal neurogenesis [62], was found to be decreased herein. Indeed, such a discrepancy may be indicative of a specific stage of suicidal individuals or distinct compensatory mechanisms. Altogether, these downregulated miRNAs support the notion of a broad impairment of post-transcriptional gene regulation in the biology of suicide.

Upregulated miRNAs included hsa-miR-32, -765, -583, -921, -30c-2, -550, -574-5p, -576-3p, -198, and -503. Several of these have been associated with schizophrenia, gliomas, ischemic stroke, or developmental disorders [63,64,65,66,67]. Of interest, both miR-574-5p and miR-198 have been linked to schizophrenia and tumor biology. The miR-574-5p is increased in blood exosomes from patients with schizophrenia and may regulate BDNF, NRG1, and DRD2, implicated in suicide risk [64]. An increase in miR-198 levels enhances temozolomide sensitivity in glioblastoma by inhibiting *MGMT*, with variants of its targets (e.g., JUN, ATF2, TAF) being associated with schizophrenia [66]. The miR-576-3p, identified as a central node in our co-expression network, has been linked to Alzheimer’s disease through circ_0005835, which acts as a “sponge” and promotes disease progression [65]. Its increase in the context of low TrkB-T1 expression further suggests an additional disruption of neurogenesis and synaptic plasticity in the suicidal cortex.

Other upregulated miRNAs could modulate cancer-related and neurovascular processes. The miR-32 acts as a tumor suppressor in glioma by targeting *ABCC4* and *EZH2* [68]. The miR-503 is induced by TGF-β1 in glioblastoma and promotes proliferation, migration, invasion, and the release of immunosuppressive factors [69]. The miR-550 is associated with hematologic malignancies through mechanisms involving the m6A machinery [70]. In the vascular domain, high miR-30c-2-3p levels in exosomes derived from macrophages within atherosclerotic plaques can exacerbate ischemic stroke by activating microglia and inhibiting SMAD2 [63]. In agreement, the miR-30c-2-3p upregulation described herein, along with simultaneous increases in chemokines and IL-1β, suggests a role in the chronic neuroinflammation of the prefrontal cortex. Moreover, miR-583, which decreased in the serum of patients with stroke and poor recovery [71], was found to be elevated in the present study, highlighting the importance of context and sample type. The miR-921 inhibits glutathione peroxidase 3 in lung cancer, impairing antioxidant capacity [72]. Otherwise, co-expression networks showed that miR-29, miR-127, miR-149, and miR-125b are central nodes among the downregulated miRNAs, while miR-576-3p, miR-493, miR-550, miR-30b, miR-16-5p, miR-181a-5p, and miR-184 are prominent among the upregulated miRNAs. Functional enrichment of their target genes indicates the participation of VEGF, PPAR, mTOR, MAPK, Wnt, and p53 signaling pathways, as well as dopaminergic synapse and long-term potentiation, all of which are key elements for emotional regulation and neuronal survival [9].

This study has certain limitations that should be considered when interpreting the findings. The analyses were conducted using publicly available datasets derived from independent cohorts and anatomically related, but come postmortem from distinct prefrontal cortical subregions (dorsolateral prefrontal cortex, BA9; and frontal cortex, BA10). As a result, direct subject-level integration of mRNA and miRNA expression profiles was not possible. Therefore, the inferred regulatory interactions represent knowledge-based in silico associations derived from curated molecular interaction databases rather than experimentally paired transcriptomic measurements along the same individuals or brain regions. As in all postmortem studies, potential confounding variables such as agonal factors, clinical heterogeneity, medication status, or comorbid psychiatric conditions cannot be fully controlled. Despite the lack of significant differences regarding postmortem interval (PMI) and brain pH between groups, as reported in the primary studies, the biological variability inherent to postmortem human tissue should be considered. Therefore, the somewhat uncertain circumstances surrounding death and tissue management restrict interpretations regarding transcriptional signatures involving stress-response genes, heat shock proteins, or immediate early genes. Nevertheless, the study presents important strengths. We analyzed independently mRNA and miRNA expression profiles derived from datasets of defined prefrontal and frontal cortical regions, applied false discovery rate correction in differential expression analyses, and restricted regulatory network construction to experimentally validated human miRNA target interactions. By integrating independently derived differential expression signatures through curated interaction databases, this approach enabled the identification of convergent molecular signals associated with the BDNF/TrkB–MAPK axis within stress-responsive transcriptional programs.

Future investigations should focus on validating the key miRNA–mRNA regulatory interactions identified herein, for example, those related to neurotrophic signaling pathways. Experimental studies examining the functional consequences of altered TrkB-T1-associated regulatory networks in astrocytes and cortical circuits may help to clarify their mechanistic relevance. Replication in larger cohorts, anatomically matched samples, and integration with cell-type–resolved or single-cell transcriptomic data could further refine the proposed regulatory framework and strengthen its translational relevance. By integrating mRNAs and miRNAs into a joint regulatory network, we identified hub genes such as *TP53*, *FOXO3*, *RELA*, *MYC*, *PTEN*, *NFKB1*, *JUN*, and *FOS*, previously linked to transcriptional networks in suicidal brains. Convergent dysregulation at mRNA and miRNA levels suggests that suicidal behavior is associated with a loss of post-transcriptional “buffering” on key stress-response and neuroimmune nodes. Several of the miRNAs identified here (miR-15b, miR-29c, miR-545, miR-373, miR-574-5p, miR-198, among others) have already been proposed as peripheral biomarkers in Alzheimer’s disease, Parkinson’s disease, schizophrenia, stroke, or cancer [47,49,52,53,57,63,64,65,66,71,72]. Their dysregulation in cortical tissue from suicide victims raises the possibility that peripheral miRNA signatures may reflect central changes associated with suicide risk.

## 4. Materials and Methods

### 4.1. Gene Expression Profile Data

To investigate transcriptomic alterations associated with suicide, publicly available datasets were retrieved from the Gene Expression Omnibus (GEO) database [73] using the following criteria: (i) post-mortem human brain tissue; (ii) case–control design comparing suicide victims and non-suicidal controls; (iii) availability of raw or processed data suitable for differential expression analysis; and (iv) sufficient sample size for statistical modeling. Based on these criteria, two independent datasets were selected and analyzed separately: GSE34120 (microRNA microarray) and GSE101521 (RNA sequencing). Demographic and clinical characteristics were obtained from the original publications associated with each dataset.

The GSE101521 dataset [9] includes postmortem dorsolateral prefrontal cortex (dlPFC; Brodmann area 9) samples from adults diagnosed with Major Depressive Disorder (MDD) who died by suicide (*n* = 21) and non-psychiatric sudden-death controls (*n* = 21) for mRNA analysis. The cohort was predominantly male, and groups were matched for age and sex. The mean age at death corresponded to mid-adulthood, and no significant differences were reported between suicide and control groups in age, postmortem interval (PMI), or brain pH. Within the same study, small RNAs sequencing was performed in nine controls and nine suicide cases; however, as no significant differentially expressed miRNAs were detected, this subset was not used for regulatory inference in the present analysis.

The GSE34120 dataset [12] comprises frontal cortex (BA10) samples from adult suicide victims (*n* = 4) characterized by reduced TrkB-T1 expression and matched non-suicidal controls (*n* = 4). Subjects were adults with comparable age ranges and a mixed sex distribution, as reported in the original publication. Brain tissue was obtained from the frontal cortex, and detailed postmortem parameters, including age, sex, PMI, and brain pH, are described in the original study. The analytical workflow consisted of three steps: (i) identification of differentially expressed miRNAs and mRNAs within each dataset; (ii) knowledge-based regulatory inference by mapping independently derived gene lists onto curated interaction databases using the program miRNet 2.0 [74]; and (iii) construction of within-dataset co-expression networks using mutual information

### 4.2. Differential Expression Analysis of miRNAs and mRNAs

Differential expression analysis was performed on the microarray data (GSE34120) using the LIMMA 3.60.6 package from Bioconductor [15]. The data were normalized using the variance-stabilizing normalization (VSN) method to stabilize variance across expression levels, followed by a log_2_ transformation. Linear modeling was then applied to estimate differential expression between the suicide and control groups. Multiple testing correction was performed using the Benjamini–Hochberg procedure [75], and microRNAs with an adjusted *p*-value of less than 0.05 were considered to be significantly differentially expressed. For RNA-seq data (GSE101521), differential expression analysis of mRNAs was conducted using the DESeq2 1.44.0 package (Bioconductor). Count data were normalized using a negative binomial generalized linear model framework, including dispersion estimation and shrinkage. Differential expression was assessed using Wald statistics, and the *p*-values were adjusted for multiple testing using the Benjamini–Hochberg method [75]. Genes with an adjusted *p*-value of less than 0.05 were considered to be significantly differentially expressed. Differential expression analyses were conducted independently for each dataset. No cross-dataset correlation analyses were performed, given that the samples originated from distinct cohorts and brain banks.

### 4.3. Heatmaps for Differentially Expressed miRNAs and mRNAs

To visualize differential expression patterns of mRNAs and miRNAs within each dataset, heatmaps were generated using the “pheatmap” function in R 3.60.6. Rows represent gene symbols (for mRNAs) or miRNA names, annotated according to miRBase. Color intensity reflects log2 fold change values between suicide and control groups, with warm colors indicating upregulation, cool colors indicating downregulation, and white representing unchanged or minimally altered expression levels.

### 4.4. Regulation Networks and Functional Enrichment

For the construction of knowledge-based regulatory inference models, miRNet 2.0 (https://www.mirnet.ca/) was used (accessed on 6 March 2026) [74]. miRNet is a network-based visual analytics platform that integrates multiple curated miRNA interaction databases and annotation resources [74]. The underlying knowledge base incorporates updated releases from major miRNA annotation and interaction repositories, including experimentally validated miRNA–mRNA interactions from miRTarBase and TarBase, supported by functional assays such as luciferase reporter assays, RT-qPCR, Western blotting, and CLIP-seq.

For this study, the organism was restricted to Homo sapiens, and only experimentally validated miRNA–target interactions were selected, limiting targets to mRNAs. Differentially expressed miRNAs and mRNAs were identified independently within their respective datasets and subsequently mapped onto curated interaction databases using miRNet to infer regulatory relationships. No direct correlation or co-expression analysis was performed, as the datasets originated from independent cohorts. Both forward (miRNA-to-target gene) and reverse (gene-to-regulatory miRNA) mappings were conducted using miRNet’s internal algorithms to identify regulatory interactions based on curated experimental evidence. Network visualization employed the Circular Tripartite layout to distinguish miRNAs, mRNAs, and transcription factors. Node size represents degree centrality (the number of connections per node). Edges represent curated regulatory interactions and were visualized as unweighted connections, without encoding edge thickness, interaction confidence, or additional quantitative metrics.

Functional enrichment analysis was conducted using KEGG pathway [76] and Gene Ontology (GO) annotation libraries [77]. Enrichment significance was calculated using a hypergeometric test, and multiple testing correction was performed using the Benjamini–Hochberg method [75]. Adjusted *p*-values (FDR < 0.05) were considered statistically significant. For the enrichment analysis, an expanded first-degree interaction network of differentially expressed genes and genes listed in miRNet was generated by selecting the organism Homo sapiens, the interaction of mature miRNAs with transcription factors, and the miRTarBase V9.0, TarBase v9.0, and miRecords databases, without selecting any tissue-specific genes. This expansion allowed the inclusion of functionally connected genes that could participate in the same biological pathways as the differentially expressed genes. Enrichment analyses were then performed using the gene set derived from this expanded network. Because the miRNA and mRNA datasets originated from independent cohorts, the resulting networks represent an in silico inference of potential regulatory interactions based on curated molecular interaction databases and independently derived differential expression signatures, rather than empirically measured biological co-expression relationships across paired samples.

### 4.5. Co-Expression Networks

Co-expression networks were constructed in R [78]. No cross-dataset correlation analyses were performed independently within each dataset (i.e., mRNA and miRNA datasets separately). After assessing the linearity of expression relationships, similarity measures were computed using mutual information to capture both linear and non-linear dependencies between expression profiles. After assessing the linearity of expression relationships, similarity measures were computed using mutual information to capture both linear and non-linear dependencies between expression profiles. After assessing the linearity of expression relationships, similarity measures were computed using mutual information to capture both linear and non-linear dependencies between expression profiles.

A similarity threshold was then applied to define statistically significant associations, and an adjacency matrix was constructed from these. The resulting network was derived from this adjacency matrix and visualized using the graph library in R. In these co-expression networks, nodes represent differentially expressed genes or miRNAs from the same dataset, and edges correspond to statistically significant co-expression relationships identified through mutual information. The networks were visualized as unweighted graphs, and no edge thickness or quantitative weighting was encoded in the final representation.

## 5. Conclusions

Our findings suggest that suicide-associated molecular vulnerability may involve coordinated transcriptional and microRNA-mediated regulatory alterations converging on neurotrophic signaling pathways within prefrontal cortical regions, including the dorsolateral prefrontal cortex (BA9) and frontal cortex (BA10). Integrative analyses revealed convergent signals related to the BDNF/TrkB–MAPK axis, highlighting the potential contribution of multilayered regulatory mechanisms to impaired synaptic plasticity and stress responsiveness.

A regionally consistent molecular signature across prefrontal cortical circuits in suicide victims, characterized by coordinated dysregulation of immune mediators, heat shock proteins, activity-dependent transcription factors, and miRNAs linked to neurodegeneration and neuroinflammation, is also supported. These patterns should be interpreted with caution, as some components, particularly heat shock proteins and immediate early genes, may also reflect agonal or perimortem responses associated with the circumstances of death. Whereas these interconnected mRNA–miRNA networks provide useful hypotheses regarding molecular processes potentially involved in suicide, further studies controlling for agonal factors and incorporating functional validation will be necessary to clarify their biological and clinical relevance.

## Data Availability

The datasets analyzed in this study are publicly available in the Gene Expression Omnibus (GEO) under accession numbers GSE34120 and GSE101521.

## Abbreviations

The following abbreviations are used in this manuscript:

ALS	Amyotrophic Lateral Sclerosis
ATF3	Activating Transcription Factor 3
BDNF	Brain-Derived Neurotrophic Factor
BA9	Brodmann Area 9
CCL2/3/4	C-C Motif Chemokine Ligand 2/3/4
CSF	Cerebrospinal Fluid
DEGs	Differentially Expressed Genes
DNAJB1	DnaJ Heat Shock Protein Family Member B1
FOXO3	Forkhead Box O3
GADD45B	Growth Arrest and DNA Damage-Inducible Beta
GEO	Gene Expression Omnibus
GO	Gene Ontology
HPA	Hypothalamic–Pituitary–Adrenal
HSP/HSPA1A/B	Heat Shock Proteins/Heat Shock Protein Family A Member 1A/1B
IL1β	Interleukin 1 Beta
JAK-STAT	Janus Kinase–Signal Transducer and Activator of Transcription
KEGG	Kyoto Encyclopedia of Genes and Genomes
LGR6	Leucine-Rich Repeat-Containing G-Protein Coupled Receptor 6
MAPK	Mitogen-Activated Protein Kinase
miRNA	MicroRNA
mRNA	Messenger RNA
mTOR	Mechanistic Target of Rapamycin
NE	Norepinephrine
NPAS4	Neuronal PAS Domain Protein 4
NR4A1	Nuclear Receptor Subfamily 4 Group A Member 1
p53	Tumor Protein p53
PTEN	Phosphatase and Tensin Homolog
TrkB-T1	Tropomyosin Receptor Kinase B (Truncated Isoform 1)
VEGF	Vascular Endothelial Growth Factor
VSN	Variance Stabilizing Normalization
